# Development and Evaluation of the Acaricidal Activity of Xantan Gum-Based Hydrogel and Polymeric Nanoparticles Containing *Achyrocline satureioides* Extract

**DOI:** 10.3390/gels10100658

**Published:** 2024-10-14

**Authors:** Rafaela Regina Fantatto, Annelize Rodrigues Gomes, João Vitor Carvalho Constantini, Camila Fernanda Rodero, Marlus Chorilli, Ana Carolina de Souza Chagas, Ana Melero, Rosemeire Cristina Linhari Rodrigues Pietro

**Affiliations:** 1 Departament of Drugs and Medicines, São Paulo State University UNESP, Rodovia Araraquara-Jaú Km 1, Araraquara 14800-903, Brazil; rrfbio@hotmail.com (R.R.F.); annelize.gomes@unesp.br (A.R.G.); joao.constantini@unesp.br (J.V.C.C.); camila.rodero@unesp.br (C.F.R.); marlus.chorilli@unesp.br (M.C.); 2 Southeast Livestock Unit, EMBRAPA-Brazilian Agricultural Research Corporation, São Carlos 13560-970, Brazil; carolina.chagas@embrapa.br; 3 Department of Pharmacy and Pharmaceutical Technology and Parasitology, Faculty of Pharmacy, University of Valencia, Avda. Vincent Andrés Estellés s/n, 46100 Burjassot, Spain; ana.melero@uv.es

**Keywords:** *Achyrocline satureioides*, *Rhipicephalus microplus*, hydrogel, xanthan gum, acaricidal activity

## Abstract

The *Rhipicephalus microplus* tick causes enormous economic losses in livestock farming around the world. Despite several promising studies carried out with plant extracts such as *Achyrocline satureioides* against this ectoparasite, a major obstacle is related to pharmaceutical presentation forms. There is no study showing xantan gum-based hydrogel and polycaprolactone nanoparticles containing *A. satureioides* extract against *R. microplus* larvae. The objective of this study was to incorporate *A. satureioides* extract to develop a nanoformulation (AScn) and a hydrogel (ASlh) and evaluate them against *R. microplus* larvae with the purpose of increasing the contact time of the extract with the larvae and improve the effectiveness. The ethanolic extracts were incorporated in polycaprolactone nanoparticles and characterized via analysis of the mean hydrodinamic diameter and polidispersity index. The xanthan gum-based hydrogel formulation was prepared with crude extract of *A. satureioides* 40 mg/mL, 0.25% xanthan gum, and 8% poloxamer, to determine the bioadhesiveness of the formulation in bovine leather and the flow rate of the formulation in the animal. The results in larvae demonstrated that when evaluated in the form of a hydrogel (ASlh), mortality was higher, with 91.48% mortality at a concentration of 20 mg/mL presenting itself as an interesting alternative for controlling this ectoparasite.

## 1. Introduction

The tick *Rhipicephalus microplus* causes huge economic losses in livestock farming worldwide. The consequences of infestations by this ectoparasite include reduced weight gain and milk production in cattle, increased veterinary expenses and, in severe cases, even death of the animal [1].

The control of *R. microplus* is mostly based on the use of conventional acaricides which can lead to the emergence of resistance and alternative approaches, including the use of animal husbandry practices, manual removal of ticks, release of sterile male hybrids, environmental management, plant species that are unfavorable to ticks, etc. [2].

Despite several promising studies carried out with plant extracts against the tick *R. microplus*, a major obstacle is related to the pharmaceutical forms of presentation. The extracts are generally evaluated in the form of a water/extract solution; however, the liquid form, due to its low viscosity, low physical–chemical stability, rapid degradation, and short residual period, is not capable of promoting long-term adhesion to the parasite, thus reducing its time of action [3,4]. Nanotechnology contributes to the physicochemical stability of formulations, and nanoencapsulation can improve the bioavailability and biological effects of active compounds, increase apparent aqueous solubility, contribute to the slow release of active compounds, and protect them against premature degradation [5,6,7]. Among the advantages, the incorporation of extracts into systems increases the solubility of the active ingredient, protects the active ingredient against hydrolysis, reduces the toxicity of the active ingredient, and increases permeation and the time of availability [8,9]. Hydrogels are three-dimensional polymeric networks formed by macromolecules interconnected via physical interactions or covalent bonds [10]. Among the main characteristics of this system are its ability to absorb large quantities of liquids and controlled release of active substances [11]. Among the polymers used to obtain hydrogels, xanthan gum stands out. It is a polysaccharide produced from simple sugars via fermentation and derives its name from the species of bacteria used, the *Xanthomonas campestris* [12].

Easy access to native plants from the Cerrado and the use of low-cost materials make a product formulated with plant extracts to be developed attractive to rural producers. Once its effectiveness is proven, as well as the cost reduction and the promotion of sustainable livestock farming, the achievements of the results will be more apparent than the inefficient and high-cost products that exist on the market. The vegetal species *Achyrocline satureioides* (Lam.) D. C., known for growing spontaneously in pastures and even being considered a “weed” [13], has already shown activity against the tick *R. microplus*. The crude ethanol extract obtained from the inflorescences of this plant species showed 100% inhibition of egg laying by the tick *R. microplus* at a concentration of 100 mg/mL and 92.3% at a concentration of 50 mg/mL. On the larvae of this same species, the extract showed 67.34% mortality at a concentration of 100 mg/mL [14]. To increase the efficacy via decreasing the concentration of the *A. satureioides* extract, this present study aimed to develop a nanoformulation and a hydrogel and evaluate them against *R. microplus* larvae.

## 2. Results and Discussion

### 2.1. Analysis of Plant Extract via HPLC-MS

**Figure 1:**
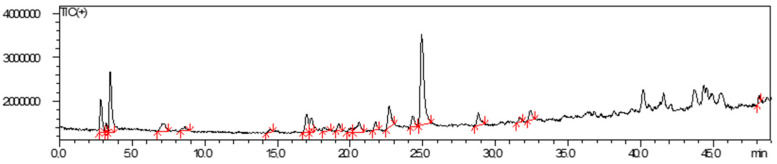
Chromatogram obtained from *A. satureioides* (ASb) inflorescences. Analysis conditions: methanol (LC-MS grade) (MeOH): MilliQ water at 1% acetic acid with a gradient of 38:100% over 45 min, 100% MeOH over 10 min and 100:32% over 7 min and a flow of 0.5 mL/min.

**Figure 2:**
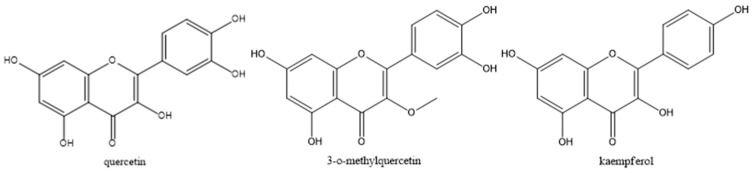
Quercetin, 3-O-methylquercetin, and kaempferol molecules found in the ethanolic extract of *A. satureioides* (ChemDraw Ultra 12.0).

The analyses performed using HPLC-MS of the plant extract are presented in the form of a chromatogram with integrated peaks (Figure 1) and a table with mass/charge (*m*/*z*) and % of two peaks (Table 1). Possible compounds such as quercetin, 3-O-methylquercetin, and kaempferol are suggested due to their mass/charge found in the literature (Figure 2).

The analyses carried out using the HPLC-MS method on the crude ethanolic extract of *A. satureioides* (AS) demonstrated the presence of quercetin, 3-O-methylquercetin, and kaempferol, corroborating other authors; however, in this extract, the presence of caffeic acid or of luteolin, commonly found in this species, was not detected [15,16,17]. Some factors may have caused this absence, such as the growing season, climate, temperature, and soil, which can alter its constituents [18]. Another aspect of the composition of the extract, related to flavonoids, especially quercetin, demonstrates that in a molecular docking study, it has inhibitory activity on larval acetylcholinesterase [19]. Generally, to obtain a high acaricidal potential, high concentrations of plant extracts such as 100 and 50 mg/mL are necessary; however, an obstacle when working with natural products is their low yield. Therefore, one way to reduce the concentrations to be evaluated and enhance their acaricidal effect is the development of formulations.

### 2.2. Mean Hydrodinamic Diameter and Polispersity Index of Nanoparticles

Table 2 presents the analysis of the mean hydrodinamic diameter and polidispersity index of AScn sample. It was observed that with the incorporation of the extracts into the nanoparticles, the ASb sample considerably decreased in diameter, suggesting an abnormality in the results. The size of the nanoparticles varied depending on the extract, with negative potential being the most common when the polymeric system with poloxamer was used. Nanotechnology studies particles with a size range of approximately 1 to 100 nm, and in the pharmaceutical area up to 1000 nm, determining their use in several applications [20]. The polydispersity index values obtained indicate homogeneity in particle size distribution. Observing the results obtained in this work, we can suggest that there were obtained nanoparticles of approximately 190 nm.

Nanotechnology has stood out for developing products with stability, greater penetration and absorption power, greater availability, and controlled release [21]; however, to guarantee these characteristics, some tests are necessary, such as particle size and the polidispersity index. The analyses carried out on the nanoparticle developed in this work according to the methodology of Greatti et al. [7].

### 2.3. Development of Hydrogel-Based Ethanolic Extract of A. satureioides

The formulation was developed with the purpose of increasing the adhesiveness of the extracts on the tick and/or animal, thus prolonging the contact time and consequently increasing the effectiveness. Thus, to determine the adhesion capacity of the formulation, the bioadhesiveness test of the formulation (bioadhesive force and bioadhesion work) and the flow rate of the formulations were determined.

#### Determination of the Bioadhesiveness of the Formulation

**Figure 3:**
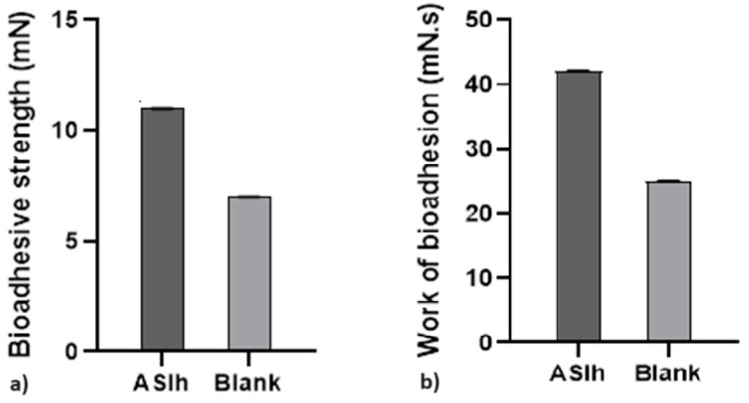
(**a**) Strength of bioadhesion of samples on bovine leather. (**b**) Bioadhesion work of the samples on the leather.

Figure 3 demonstrates the adhesive potential of the of loaded-hydrogel formulation with ethanolic extract of *A. satureioides* (ASlh) on bovine leather.

The bioadhesion on the formulation was approximately 1.7 times higher in comparison with the blank. The extract contributed to increasing the bioadhesion of the hydrogel in both tests.

The formulations are also considered pharmaceutical forms capable of improving the effectiveness of plant extracts as due to their better adhesiveness, the compound remains on the target for longer and compounds that promote these characteristics are normally used. Poloxamer 407 has excellent thermosensitive gelling properties, however, it is poor in terms of mucoadhesive properties due to its high permeability to water, and the addition of a compound that has this function is recommended [22]. In this present work, we chose to use xanthan gum, because this gum is a biodegradable, cheap and easily acquired material that has the ability to form gel in the presence of water [23].

Xanthan gum, produced by several types of Xanthomonas bacteria, is a natural polymer composed of glucuronic acid and mannose, widely used in various foods, lotions, shampoos, and dermatological items. Furthermore, due to its physicochemical characteristics, xanthan gum is being used to develop and expand drug delivery systems [24]. Due to its chemical composition and high molecular weight, xanthan gum has exceptional pseudoplasticity, thickness, and rheological capabilities, and is extremely stable to acids, alkalis, and heat [25]. Xanthan gum is frequently used in the food, textile, pharmaceutical and oil recovery industries as a rheological modifier, stabilizer, and emulsifier, as well as a biodegradable, cheap, non-toxic, and bioadhesive pharmaceutical excipient [26].

There are several studies showing the use of hydrogels based solely on xanthan gum or cross-linked with other polymers [27,28]. Xanthan gum, the control release agent included in the formulations, is known to have the ability to hydrate very rapidly in addition to exhibiting a great swelling index compared with other gums [29]. Xanthan gum hydrogels have been applied as drug carriers and controlled drug release systems, allowing the controlled delivery of different medications such as caffeine, levofloxacin, and ibuprofen [30,31,32]. Xanthan gum is being used with plant extracts in the area of food preservation where films are generated [33] and to preserve juice fruits [34]. The adhesiveness of a formulation is determined using tests to determine the bioadhesiveness of the formulation (bioadhesive strength and bioadhesion work). When evaluating adhesiveness, it was observed that when compared with the blank, the ASlh formulation showed lower bioadhesion.

### 2.4. Determination of the Flow Speed of the Formulations

**Figure 4:**
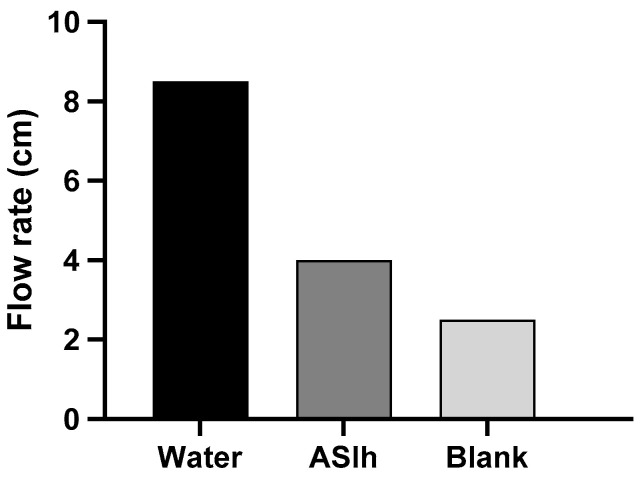
Flow distance for hydrogel formulation containing extract (ASlh), blank (xanthan gum and poloxamer), and water in contact with the bovine leather.

**Figure 5:**
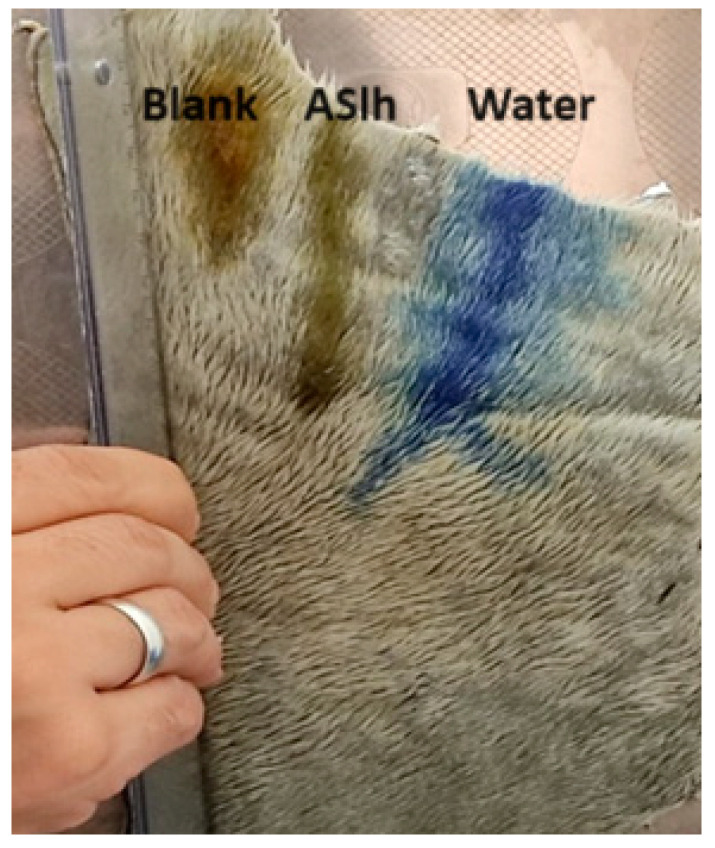
Test to determine the flow of formulations on bovine leather (Rafaela Fantatto).

The determination of the flow rate of the formulations was carried out on fresh bovine leather and the distances (in cm) that the formulation flowed were measured in a time of 2 min and the results obtained are presented in Figure 4 and Figure 5.

The higher flow distance was obtained by water, followed by the hydrogel formulation containing *A. satureioides* extract, and finally by blank, which proved to be restricted to a small distance covered during the test time. The effect of the *A. satureioides* extract was to promote the destructuring of polymeric matrices. In Figure 5, there is a picture of the flow distance in the bovine leather.

In addition to the adhesiveness tests of the formulation carried out by the bioadhesive strength and bioadhesion work, the flow speed of the formulations was also analyzed, in which the ASlh formulation flowed faster, 6 cm in 2 min, much closer to the water that drained 8 cm in the same period of time. The association of active substances, such as those found in crude plant extracts, can impair the stability and alter the characteristics of a formulation [35]. However, even losing part of its adhesiveness, it still presents better adhesion and flow time compared to the extract solubilized only in water and flow speed of the formulations.

### 2.5. Mortality of R. microplus Larvae

Following the methodology proposed by the FAO [36], the nanoemulsion and the formulation containing ASlh were also evaluated. In Table 3, we can see the results of larvae mortality against the nanoformulation.

The impregnated paper test for ethanolic extract nanoparticles of *A. satureioides* did not demonstrate a high larval mortality rate. However, the same test carried out for the xanthan gum-based hydrogel formulation showed a much better result (Table 4).

It can be noted that at the highest evaluated concentration of the formulation we obtained a high mortality rate of larvae (91.48% at the 20 mg/mL of extract concentration) and that even with half the concentration (10 mg/mL), the mortality rate, in relation to the highest, reached 78.13%. The lowest concentrations showed 12.94% and 20.81% in the mortality rate, respectively, in relation to the highest concentration evaluated. These results showed a significant improvement in acaricidal activity when the extract was incorporated in the hydrogel, which can also be verified by the low activity of the extract without being incorporated into the hydrogel.

The mortality rate of the nanoparticle was low, while the formulation had a better mortality rate, reaching 20.88 times higher, presenting 91.48% mortality. Studies carried out by Fantatto et al. (2022) demonstrated that the ethanolic extract of *A. satureioides* presented 67.34% mortality at a concentration of 100 mg/mL [14], and another study developed by Fantatto et al. (2022) [37] evaluated the ethanolic, acetate, and hexane extracts from inflorescences of this same species without being included in formulations and the mortality percentages found at a concentration of 20 mg/mL were 2.43, 0.42 and 1.33%, respectively, which when compared with our results, shows that the xanthan gum-based hydrogel formulation demonstrated a much better result. Xanthan gum has been used in solutions and has been widely studied not only for its commercial importance but also for its characteristic of not undergoing conformational transition due to changes in temperature, ionic strength, pH, and polymer concentrations [38]. This polymer has been approved by the Food and Drugs Administration (FDA) for use in food since 1969 [39] and is also used in oral and topical pharmaceutical formulations and cosmetic products and is generally considered non-toxic or non-irritating at the levels used as an excipient [38]. It can be observed that although poloxamer and xanthan gum do not have toxicity, both presented a mortality rate, which may have been caused by obstruction of the spiracles, impairing gas exchange and leading to the death of the larvae [40], enhancing the effect of the formulation and acting as excellent adjuvants for acaricides.

## 3. Conclusions

The extract of *A. satureioides* proved to be potentially attractive to aid in the control of the *R. microplus* cycle in both formulations developed. This was reinforced when formulated in a hydrogel based on xanthan gum, as it maintained the property of killing larvae, as well as the application on bovine leather, where it remained in contact for a certain time and was thus able to exert its acaricidal effect. In addition, among the flavonoids, quercetin is known to present in vitro inhibition of acetylcholinesterase and mortality of tick larvae, being a potential acaricide. As we found no studies demonstrating the formulation of a hydrogel based on xanthan gum containing *A. satureioides* extract against *R. microplus* larvae, we consider these results very important for the control of *R. microplus* and for the future development of a product with low economic cost, highlighting the importance of this article.

## 4. Material and Methods

### 4.1. Plant Material

The plant material of *A. satureioides* was obtained from the CPQBA Medicinal Plants Collection, where it was collected in the city of Campinas-SP under coordinates 22°48′ S; 47°07′ W and had been registered and authorized for use by SISGEN under registration number AD73F75.

### 4.2. Production and Fractionation of Plant Extracts

Plant material was dried in an oven with air circulation at 40 °C and later ground in a knife mill. Ethanol extracts were obtained from aerial parts of *A. satureioides* using the method of maceration 1:10 in absolute ethanol. After 7 days, the contents were filtered, rotaevaporated and stored in an amber bottle.

### 4.3. Extract Analysis Using HPLC-MS

The extract was analyzed using HPLC-MS on Shimadzu equipment with an LC-20AD pump and a CTO-10AS VP column oven coupled to a single quadrupole mass spectrometer as an analyzer (LC-DAD_MS-2020 QP Shimadzu/Bruker, Kyoto, Japan), with an electrospray ionization (ESI) source. An ACE 3 C18 column (150 mm × 4.6 mm, 3 µm particle size) with an ACE3 C18 analytical pre-column was used for separation. The compounds were eluted with methanol (MeOH, LC-MS grade), MilliQ water to 1% acetic acid with a gradient of 38:100% over 45 min, and 100% MeOH over 10 min and 100:32% over 7 min at a flow rate of 0.5 mL/min. The nitrogen flow (drying gas for solvent evaporation) was 15 L/min. The potential for the electrospray capillary was +4.50 kV and Full Scan was used in positive and negative mode (*m*/*z* 100–700) with a potential of 1.40 kV and a capillary temperature of 250 °C. The stock solution of the ethanolic extract was injected at 0.25 mg/mL, with an injection of 5 µL through an automatic injector (SIL-20A XR, Shimadzu, Kyoto, Japan). The extract was dissolved in 100% MeOH for injection. For quantification, the relative area percentages of all peaks obtained in the chromatograms were used and the ones with the highest area percentage of each sample were chosen.

### 4.4. Development of Polycaprolactone (PCL) Nanoparticles

The nanoparticles were developed using the classical nanoprecipitation methodology adapted by Greatti et al. (2020) [7]. The polycaprolactone polymer together with the ethanolic extract of *A. satureioides* at a concentration of 20 mg was dissolved in 50 mL of acetone, and the surfactant Poloxamer 407 (polyethylene glycol) at a concentration of 0.5% was dissolved in 100 mL of deionized water as the aqueous phase of the formulation. The organic phase was immersed in the aqueous phase with stirring in a laminar flow hood for approximately 16 h for total evaporation of the organic solvent. The final volume was 50 mL and was denominated AScn (*Achyrocline satureioides* crude nanoparticles) in this study. The blank nanoparticle was developed using the same procedures but without the inclusion of *A. satureioides* extract.

### 4.5. Mean Hydrodinamic Diameter (MHD) and Polidispersity Index (PI) of Nanoparticles

MHD and PI analyses were performed using a Zetasizer Nano NS (Malvern Instruments, Malvern, UK). The nanoparticles were diluted 100× in distilled water. The samples were placed in the analysis chamber so that the laser beam passed through the entire dispersion. The system temperature was maintained at 25 °C, the laser wavelength was 532 nm, and the refractive index was set to the index observed for each sample analyzed. Six determinations of the MHD and PDI of the nanoparticles of each sample were performed, with a total duration of 5 min. The same procedure was applied to the blank nanoparticles.

### 4.6. Production of Ethanolic Extract of A. satureioides Loaded-Hydrogel (ASlh)

The formulation was prepared using an ethanolic extract of *A. satureioides* at a concentration of 20 mg/mL. This extract was incorporated into a solution containing 0.25% xanthan gum and 8% poloxamer (ASlh). The controls were blank (only xanthan gum and poloxamer in the concentrations above) and water with 10% methylene blue dye was used as a negative control. The formulation was prepared as follows: the ethanolic extract of *A. satureioides* was weighed at an initial concentration of 40 mg/mL and dissolved in 1 mL of distilled water with 0.5% commercially available xanthan gum. A second solution containing distilled water and 16% poloxamer was prepared and dissolved in an ice bath. After both solutions were prepared, they were incorporated into each other, resulting in half the amount of xanthan gum and poloxamer, i.e., reaching 0.25% xanthan gum and 8% poloxamer.

### 4.7. Determination of the Bioadhesiveness of the Loaded-Hydrogel

The raw bovine leather was obtained commercially, washed, cut into small squares of 5 cm on each side and frozen at −80 °C. On the day of the experiment, the leather was thawed and washed in running water. The bioadhesive strength between the leather and the hydrogels was evaluated via the detachment test using a texture analyzer described by Carvalho et al., modified [41].

The leather was attached to the lower end of the analytical probe (diameter 10 mm) with a rubber ring. The formulation samples were placed in shallow cylindrical vessels and the leather-coated analytical probe was lowered at a constant speed (1 mm/s) until it reached the surface of the sample. The leather was kept in contact for 60 s and no force was applied during this interval. Then, the leather-coated analytical probe was pulled upwards (0.5 mm/s) until the contact between the surfaces was broken. The bioadhesive strength of the formulations was the measurement of the maximum separation force as the resistance to probe withdrawal, which reflects the bioadhesion characteristic. Five replicates were performed at 32 °C.

### 4.8. Determination of the Flow Rate of the Loaded-Hydrogel

To determine the flow rate of the formulation in the animal, a test was developed using raw bovine leather. A sample of approximately 20 cm of bovine leather was cleaned to remove residues, washed in distilled water and placed on a heating plate at a temperature of 38 °C and an inclination of 60 °C. For each formulation, 200 μL of, namely, ASlh, blank (only xanthan gum and poloxamer in the same concentrations), and water with 10% methylene blue dye were applied to the upper part of the leather and the distance traveled (in centimeters) by the samples of the formulations in 2 min was measured using a ruler.

### 4.9. Mortality of Larvae in Patch Test on Impregnated Paper

The sensitivity tests of *R. microplus* larvae were performed according to the technique recommended by the FAO (1971) [36]. In this methodology, approximately 100 larvae from engorged females collected at Embrapa Pecuária Sudeste were placed on filter paper measuring approximately 10 × 8 cm, impregnated with different concentrations of the samples or control. Each of these impregnated papers was folded to form a “sandwich” and sealed with plastic clips. The envelopes were stored in a BOD (Biochemical Oxygen Demand) incubator at 27 °C and 80% relative humidity, in triplicate. The reading was taken after 48 h of incubation with the help of a vacuum compressor, adapted with a pipette, differentiating live and dead larvae.

### 4.10. Statistical Analysis

All statistical analyses were conducted using the SPSS statistical software (version 24, IBM Corp., Armonk, NY, USA). Analysis of variance, Tukey test, and standard deviation calculation were performed for the results obtained in the tests. Pairwise comparisons between specific treatment groups were performed using independent samples *t*-tests. The significance level for all statistical tests was set at *p* < 0.05.

## Figures and Tables

**Table 1:** Retention time and mass/charge obtained via HPLC-MS analysis of compounds belonging to the crude ethanolic extract of *A. satureioides*.

Achyrocline satureioides (AS)					
Retention Time	*m*/*z*	[M]+		Possible Compound	
					% Peak
2.84	207 193 179 165				7.21
3.17	104 137				1.44
3.47	205 183 118 153				14.51
7.13	449 471	448	[M + H]^+^	Derivative of kaempferol	3.85
8.62	449 471	448	[M + H]^+^	Derivative of kaempferol	1.44
14.49	479 501	478	[M + H]^+^	Phyllirin	0.91
17.00	287 309	486	[M + H]^+^		5.28
17.32	465 487	464	[M + H]^+^	Isoquercetin	4.22
18.26	449 471	448	[M + H]^+^		1.31
19.22	479 501	478	[M + H]^+^		1.87
20.06	465 487	464	[M + H]^+^	Derivative of isoquercetin	1.29
20.60	449 471	448	[M + H]^+^		4.41
21.72	479 501	478	[M + H]^+^		2.01
22.70	303 325	302	[M + H]^+^	Quercetin	6.44
24.31	287 309	286	[M + H]^+^		3.24
24.93	317 339	316	[M + H]^+^	3-O-methylquercetin	31.28
28.82	287 309 301	286	[M + H]^+^	4,2′,4′-trihydroxy−6′-methoxychalcone	4.03
31.70	617 639	616	[M + H]^+^		1.17
32.46	315 337	314	[M + H]^+^		2.92
43.76	425 403 453	402	[M + Na]^+^		
45.02	467 445 320	444	[M + Na]^+^		
45.28	439 417	416	[M + Na]^+^		
48.16	437 459	436	[M + H]^+^		1.18

**Table 2:** Determination of the mean hydrodinamic diameter and polydispersity index (PDI) using Dynamic Light Scattering (DLS) of nanoparticles (AScn).

Sample	Mean Hydrodinamic Diameter	PDI
Blank	189.31 ± 6.55	0.13 ± 0.04
AScn	192.33 ± 4.35	0.044 ± 0.023

**Table 3:** Percentage of mortality of *R. microplus* larvae in contact with paper impregnated with nanoparticles of ethanolic extract of *A. satureioides* (AScn).

Concentration (mg/mL)	% Mortality
20	4.38 ± 7.1 ^a^
Negative Control (water)	0 ± 0 ^b^

The Tukey test at 5% probability level (*p* < 0.05) was applied and the standard deviation was calculated. The means followed by the same letter do not differ statistically from each other. The negative control was water.

**Table 4:** Percentage of mortality of *R. microplus* larvae in contact with paper impregnated with ethanolic extract formulation of *A. satureioides* with ethanolic extract formulation of *A. satureioides*.

Concentration (mg/mL)	% Mortality
Loaded-Hydrogel ASlh	
20	91.48 ± 14.7 ^a^
10	71.48 ± 2.1 ^b^
5	11.84 ± 8.5 ^d^
2.5	19.04 ± 2.7 ^c^
Negative Control	0.50 ± 8.9 ^f^
Control Pol 8	20.89 ± 4.4 ^c^
Control xanthan gum 0.25	4.42 ± 3.9 ^e^

The Tukey test at 5% probability level was applied and the standard deviation was calculated. The means followed by the same letter do not differ statistically from each other. Negative control: water; Control Pol 8: poloxamer in water; Control xanthan gum: only xanthan gum in water.

## Data Availability

The raw data supporting the conclusions of this article will be made available by the authors on request.
